# Effects of Gait Treatment With a Single-Leg Hybrid Assistive Limb System After Acute Stroke: A Non-randomized Clinical Trial

**DOI:** 10.3389/fnins.2019.01389

**Published:** 2020-01-22

**Authors:** Hiroki Watanabe, Aiki Marushima, Hideki Kadone, Tomoyuki Ueno, Yukiyo Shimizu, Shigeki Kubota, Tenyu Hino, Masayuki Sato, Yoshiro Ito, Mikito Hayakawa, Hideo Tsurushima, Tomoya Takada, Atsuro Tsukada, Hiroyuki Fujimori, Naoaki Sato, Kazushi Maruo, Hiroaki Kawamoto, Yasushi Hada, Masashi Yamazaki, Yoshiyuki Sankai, Eiichi Ishikawa, Yuji Matsumaru, Akira Matsumura

**Affiliations:** ^1^Center for Cybernics Research, University of Tsukuba, Tsukuba, Japan; ^2^Department of Neurosurgery, Graduate School of Comprehensive Human Sciences, University of Tsukuba, Tsukuba, Japan; ^3^Department of Neurosurgery, Faculty of Medicine, University of Tsukuba, Tsukuba, Japan; ^4^Department of Rehabilitation Medicine, University of Tsukuba Hospital, Tsukuba, Japan; ^5^Department of Orthopaedic Surgery, Faculty of Medicine, University of Tsukuba, Tsukuba, Japan; ^6^Department of Neurosurgery, Kennan Hospital, Tsuchiura, Japan; ^7^Department of Neurosurgery, Kobari General Hospital, Noda, Japan; ^8^Department of Biostatistics, Faculty of Medicine, University of Tsukuba, Tsukuba, Japan

**Keywords:** Hybrid Assistive Limb, acute stroke, independent walking, Functional Ambulation Category, gait treatment

## Abstract

We hypothesized that a single-leg version of the Hybrid Assistive Limb (HAL) system could improve the gait and physical function of patients with hemiparesis following a stroke. In this pilot study, we therefore compared the efficacy of HAL-based gait training with that of conventional gait training (CGT) in patients with acute stroke. Patients admitted to the participating university hospital were assigned to the HAL group, whereas those admitted to outside teaching hospitals under the same rehabilitation program who did not use the HAL were assigned to the control group. Over 3 weeks, all participants completed nine 20 min sessions of gait training, using either HAL (i.e., the single-leg version of HAL on the paretic side) or conventional methods (i.e., walking aids and gait orthoses). Outcome measures were evaluated before and after the nine training sessions. The Functional Ambulation Category (FAC) was the primary outcome measure, but the following secondary outcome measures were also assessed: National Institutes of Health Stroke Scale, Fugl–Meyer Assessment (Lower Extremity), comfortable walking speed, step length, cadence, 6-min walk distance, Barthel Index, and Functional Independence Measure. In total, 22 post-stroke participants completed the clinical trial: 12 in the HAL group and 10 in the CGT group. No serious adverse events occurred in either group. The HAL group showed significant improvement in FAC after nine sessions when compared with the CGT group (*P* = 0.014). However, secondary outcomes did not differ significantly between the groups. Our results demonstrate that HAL-based gait therapy may improve independent walking in patients with acute stroke hemiplegia who are dependent on ambulatory assistance. A larger-scale randomized controlled trial is needed to clarify the effectiveness of single-leg HAL therapy.

**Clinical Trial Registration:** UMIN Clinical Trials Registry, identifier UMIN000022410.

## Introduction

Stroke is a serious and disabling health problem that is common worldwide (Langhorne et al., 2011). Approximately 90% of patients who suffer a stroke will have persistent neurological motor deficits that cause disability and handicap (Hesse and Werner, 2003). In Japan, stroke is the third leading cause of death and the second leading cause of social care needs (Ministry of Health, Labour and Welfare, 2016, 2018). Given that one-third of survivors will only achieve a poor functional outcome 5 years after a stroke (Barker-Collo et al., 2010; Takashima et al., 2017), it is unsurprising that stroke-related problems place a serious burden on both patients and their families. However, effective early treatment and rehabilitation can significantly improve outcomes (Rigby et al., 2009).

The recovery of gait and independent walking are common goals following a major stroke (Dobkin, 2005; Peurala et al., 2009), and the intensity of rehabilitation therapy for the arms and legs is known to be positively correlated with motor outcomes after stroke (Kwakkel et al., 1999). Given that the recovery of walking function mainly occurs within the first 11 weeks after a stroke (Jørgensen et al., 1995), active and intensive gait treatment should start from the acute stage. Since the 1990s, automated or robot-assisted motor rehabilitation has emerged as a viable aid in this process (Hesse et al., 2003). Indeed, a recent systematic review of the Cochrane database suggested that electromechanical-assisted gait training in combination with physiotherapy was more likely to produce independent walking after a stroke than gait training without these devices. Moreover, most benefit was seen in the first 3 months after stroke and in those unable to walk (Mehrholz et al., 2017).

The Hybrid Assistive Limb (HAL) robotic suit is the world’s first cyborg-type wearable device for supporting, improving, and expanding a wearer’s physical functions based on the detection of bio-electrical signals (BES) detected on the skin surface when a wearer tries to generate muscle force (Lee and Sankai, 2005; Suzuki et al., 2007; Sankai et al., 2014). HAL treatment is based on the principles of the interactive biofeedback hypothesis. It uses a hybrid control system composed of two cybernic control modes (voluntary and autonomous) (Kawamoto et al., 2009). Once activated by the wearer’s BES, the voluntary control mode provides physical support and actions through voluntary intention (Kawamoto and Sankai, 2005). For example, the wearer’s desire to move his or her legs causes the transmission of a signal from the brain to the peripheral nerves, muscles, and skin. However, patients with stroke-induced hemiparesis cannot move their extremities, and in such patients, HAL can support movement by utilizing very weak BES on the skin surface to activate HAL autonomously (Sankai and Sakurai, 2018). This movement and exercise may enhance recovery of the impaired neuronal network, while interactive biofeedback may further promote appropriate reorganization of the neuronal network (Morishita and Inoue, 2016; Sankai and Sakurai, 2018). The single-leg version of the HAL is a new wearable robotic device that has been designed to be used by people with hemiplegia.

To date, there has been no study comparing HAL and conventional therapies on patient outcomes after acute stroke. We therefore wanted to compare the effects of gait treatment with a single-leg version of HAL against that of conventional gait training (CGT) in patients with acute stroke.

## Materials and Methods

### Objective

The purpose of this pilot clinical study was to investigate whether motion assistance during gait treatment, using the single-leg version of HAL, could produce early improvements in independent walking after acute stroke compared to CGT.

### Design and Setting

This is a non-randomized, non-blinded clinical trial. Patients hospitalized at the University of Tsukuba Hospital were assigned to the HAL group. Patients hospitalized at Kennan Hospital, Kobari General Hospital, Tsukuba Memorial Hospital, or Ibaraki Seinan Medical Center Hospital were assigned to the CGT group. Patients in the HAL group received gait training with HAL, whereas those in the CGT group received gait training without HAL. Training was provided thrice weekly for 20 min per day over a 3-week period. This led to nine sessions each during the study.

### Types of Participants

Patients admitted to one of five hospitals (University of Tsukuba Hospital, Kennan Hospital, Kobari General Hospital, Tsukuba Memorial Hospital, and Ibaraki Seinan Medical Centre Hospital) with acute-onset stroke between September 2016 and March 2019 were invited to participate in this study. The targeted numbers of cases were 20 and 30 patients in the HAL and CGT groups, respectively.

### Inclusion Criteria

All of the following inclusion criteria needed to be met:

• Hemiparesis due to unilateral ischemic or hemorrhagic acute stroke.
• Age 40–80 years.
• A time since stroke onset within 7–14 days.
• A score of 1 or 2 on the Functional Ambulation Category (FAC).
• Likely to survive or persist with the intervention for at least 3 weeks.
• A score of >4 on FAC before stroke.
• Ability to consent (using a surrogate to sign a form if handwriting is difficult).
• Suitable for HAL (only in the HAL group).

### Exclusion Criteria

Patients meeting any of the following criteria were excluded:

• Difficulty performing voluntary limb movement due to altered consciousness.
• Patient had another condition, such as severe cardiac disease or musculoskeletal problems, that could limit treatment using HAL.
• Investigator or sub-investigator deemed the patient inappropriate for inclusion in the clinical trial (i.e., patients with malignant tumors that have not been completely cured, bleeding tendencies, and/or severe mental illness).
• Patient received magnetic stimulation, electrical stimulation, or neuromodulation therapy before inclusion in the study.

### Treatments

#### Gait Treatment Using HAL and CGT

The HAL group received gait treatment using a single-leg version of HAL. To prevent falls, all patients were harnessed in a mobile suspension system (All-In-One Walking Trainer, Ropox A/S, Denmark). Distance and walking speed depended on the patient’s tolerance. The physical therapist gradually increased the training intensity by changing the speed and duration of walking based on patient fatigue and endurance. The physical therapist provided verbal instructions to achieve a symmetric gait according to the ability of each patient. Specifically, the treatment aimed to swing the paretic leg outward during the swing phase and extend the hip joint during the stance phase. The physical therapist provided real-time feedback to the patient based on the BES and floor reaction force data during gait treatment. Gait orthoses were allowed. The heart rate, blood pressure, and ratings of subjective exercise intensity (Modified Borg scale) were monitored during walking and after each 20-min treatment. The duration of each gait treatment session, including the rest time, was 20 min. Patients participated in three sessions per week with a maximum of one session per day for a total of nine sessions over a 3-week period. We measured the actual walking time (i.e., non-rest time) and distance in each 20-min session. The intervention goal was to improve independent walking, as characterized by improved walking speed, endurance, postural stability, and symmetry (Watanabe et al., 2014). The cybernic voluntary control mode was mainly used; however, for patients who could not achieve motion with this mode, the cybernic autonomous control mode was used until they became familiar with the voluntary control (Kawamoto and Sankai, 2005; Kawamoto et al., 2009). CGT in the control group, was carried out without the HAL system by the physical therapist in charge of the patient, but otherwise using the same methodology.

#### Single-Leg HAL Therapy

**FIGURE 1:**
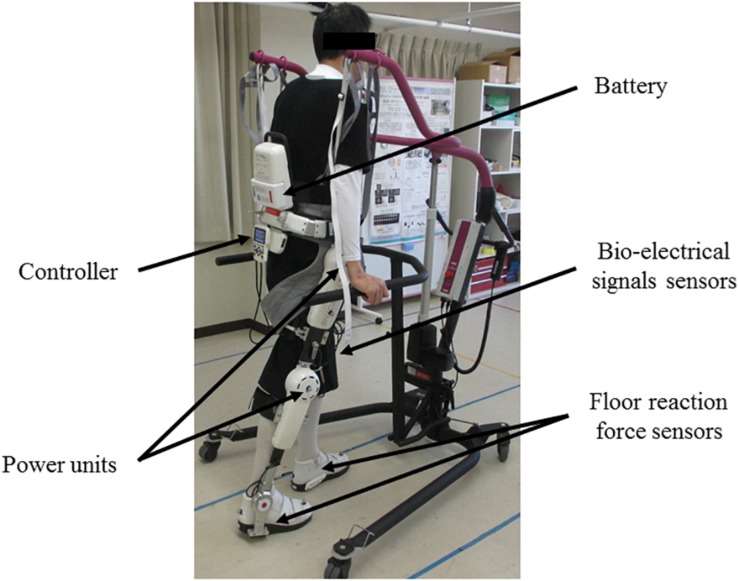
Gait treatment using single-leg version of HAL on the paretic side. The HAL is a battery-powered exoskeleton type robot. The physical therapist adjusts the belt and brings the wearer in close contact with the HAL. The controller of the HAL drives the power units based on the information of the bio-electrical signals sensors and the floor reaction force sensors to support the walking motion.

**FIGURE 2:**
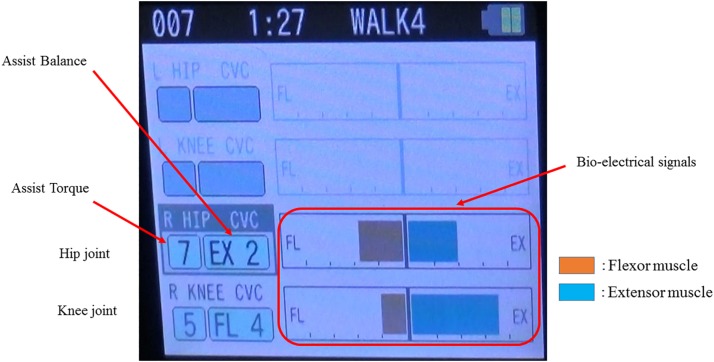
Example of the bio-electrical signals on the paralyzed side during gait treatment with HAL. The physical therapist adjusts the assist torque and assist balance based on the information of the bio-electrical signals. In this case, the assist torque of the hip joint is 7, knee joint is 5, and walking assistance is performed with the hip extension and knee flexion being dominant.

**FIGURE 3:**
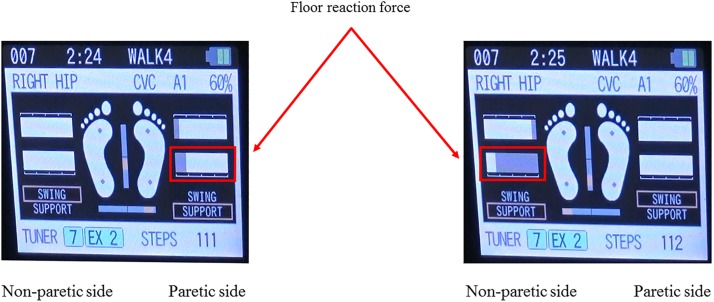
Example of the floor reaction force on the both leg during gait treatment with HAL. The floor reaction force during gait treatment with HAL can be calculated from the floor reaction force sensors built into the HAL shoes, and visual feedback can be provided to the physical therapist and the patient in real time on the controller screen as shown. This figure shows an example of the floor reaction force in the paretic and non-paretic stance phase, and it can be seen that the floor reaction force on the paralyzed side is smaller than that on the non-paralyzed side.

The single-leg version of HAL (HAL-ML05) has been described in detail in previous reports (Kawamoto et al., 2009). The device is composed of an exoskeletal frame, power units, a battery, a controller, BES sensors, and floor reaction force sensors, together with belts to secure the waist, thigh, and lower leg (Figure 1). The weight of this HAL is approximately 9 kg. Electrodes are attached on the surface of the wearer’s skin over the rectus femoris, gluteus maximus, vastus lateralis, and biceps femoris muscles to detect the nerve and muscle action potentials as BES (Figure 2). The single-leg HAL system assists hip and knee joints with flexion and extension by a hybrid control system consisting of cybernic voluntary and autonomous control (Lee and Sankai, 2005). The voluntary control system provides harmonious support based on BES, reflecting the wearer’s voluntary intentions (Kawamoto and Sankai, 2002), with the gain in assistive torque at each joint in response to the BES being controlled by a physical therapist. The autonomous control system provides torque autonomously according to the walking pattern based on input from the floor reaction force (Figure 3; Kawamoto and Sankai, 2005). These mechanisms enable the HAL to coordinate an appropriate level and timing of torque to assist with hip and knee joint motion.

#### Permitted and Prohibited Concomitant Treatments

Patients in both the groups were prohibited from participating in robot-assisted training before and during the study period. HAL-based gait treatment was considered a component of physical therapy. The content and amount of physical therapy received before study participation were not specified. Patients in both the groups participated in conventional physical therapy, including muscle and endurance strengthening and conventional over-ground gait training. No patient in either group participated in robot-assisted gait training before or during the study period. The total duration of physical therapy during the 3-week intervention protocol was limited to 16 h. However, other routine aspects of physical therapy were permitted. In addition to normal exercise therapy (i.e., range of motion, muscle and endurance strengthening, and cooperative exercise), we permitted voluntary promotion exercises, lower limb stretches, basic motion exercises, and conventional over-ground gait training. No limitations were placed on the amount or content of intervention permitted by occupational and speech therapists.

### Outcomes

#### Primary Outcome Measure

The primary outcome measure was FAC. This primary outcome was selected because a feasibility and safety study has reported the most significant improvement in this variable when compared with other outcome measures (unpublished data). FAC was evaluated by two physical therapists. Additionally, a physical therapist evaluated the appropriateness of the environment and evaluation measurement method and ensured implementation according to the manual during each gait assessment conducted at an external hospital. The gait were filmed, and assessed by a third party who was not involved in the study to confirm the correct implementation.

#### Secondary Outcome Measures

The following secondary outcomes measures were also assessed: National Institutes of Health Stroke Scale score, Fugl–Meyer Assessment of Lower Extremity, comfortable walking speed, step length, cadence, 6-min walk distance, Barthel Index, and Functional Independence Measure (total, motor scores, and cognitive scores).

### Safety Outcome Measurement

Safety was examined through adverse events, which were defined as any event clearly related to HAL or CGT. If an adverse event occurred, the investigator responded appropriately and immediately, taking care to describe the episode consistently in the medical record and a case report. Researchers recorded any unfavorable symptoms as adverse events using a case report form during the study period. A serious adverse event was considered when it led to death or threatened life, required hospitalization or extension of hospitalization for treatment, and resulted in persistent or noticeable dysfunction or treatment failure.

### Statistical Analysis

We tested differences in the baseline variables between the HAL and CGT groups by the Fisher exact test for categorical data and by the Mann–Whitney *U* test used for continuous data. The outcome measures in each group compared before and after gait training by the Wilcoxon signed-rank test; the Mann–Whitney *U* test was also used to compare the amount of change between both groups. All statistical analyses will be conducted using IBM SPSS version 24.0 (IBM Corp., Armonk, NY, United States). Statistical significance was set at *p* < 0.05.

### Ethics Approval and Consent to Participate

The study protocol was designed according to tenets of the Declaration of Helsinki and relevant ethical guidance for clinical research. All patients recruited from the four participating hospitals provided written informed consent after learning about the study aims and design. The ethics committee of the University of Tsukuba Hospital approved this study (H27-257), and the protocol has been registered with the UMIN Clinical Trials Registry (UMIN000022410).

## Results

**FIGURE 4:**
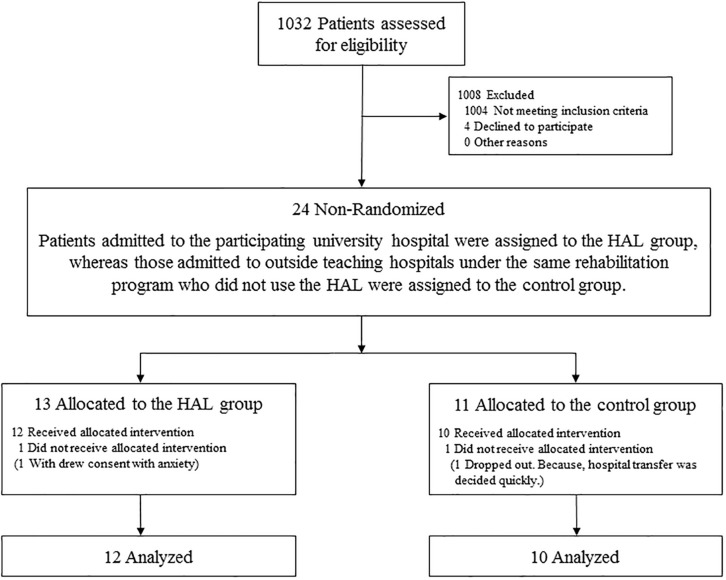
Flowchart of patient participation.

In total, 24 patients suffering from acute stroke participated in this study (Figure 4). Among the 13 patients allocated to the HAL group, one withdrew consent because of a high degree of stooped back in contact with the lumbar frame of HAL that caused anxiety. All remaining patients were ultimately able to use the cybernic voluntary control (one patient required cybernic autonomous control at the start of the intervention only). Among the 11 patients allocated to the CGT group, one dropped out because hospital transfer was decided quickly and the principal investigator agreed that it was clinically appropriate. Finally, 22 patients completed this study (Table 1). This study was terminated at the end of the indicated period before the target number of cases was reached. No serious adverse events occurred in either group. One patient in the HAL group reported pain in the contralateral (i.e., non-paralyzed) upper and lower limbs. This patient did not develop fever or inflammatory symptoms, and the pain disappeared immediately after follow-up. No patient in either group experienced orthostatic hypotension, scratches, or falls. These findings suggest that this protocol can be safely implemented.

**TABLE 1:** Demographic characteristics of patients who completed study protocol.

	**HAL group (*n* = 12)**	**Conventional group (*n* = 10)**	***P***
Age	59.1 ± 11.1	64.3 ± 8.4	0.283^a^
Sex, men/women	5/7	6/4	0.670^b^
Height (cm)	160.1 ± 12.8	160.5 ± 10.8	0.821^a^
Weight (kg)	61.7 ± 14.4	60.7 ± 8.7	0.872^a^
Type of stroke, ischemic/hemorrhagic	7/5	6/4	1.000^b^
Side of paresis, right/left	7/5	5/5	1.000^b^
Hypertension, yes/no	7/5	8/2	0.381^b^
Diabetes mellitus, yes/no	1/11	3/7	0.293^b^
History of cardiac disease, yes/no	2/10	1/9	1.000^b^
Hyperlipidemia, yes/no	0/12	1/9	0.455^b^
MMSE	25.9 ± 3.3	26.4 ± 4.3	0.539^a^
Attention disorder, yes/no	4/8	5/5	0.666^b^
Aphasia, yes/no	2/10	1/9	1.000^b^
Unilateral spatial neglect, yes/no	1/11	1/9	1.000^b^
Surgery in acute treatment, yes/no	1/11	1/9	1.000^b^
Intravenous t-PA, yes/no	1/11	1/9	1.000^b^
Days of rehabilitation from stroke onset before participating in the study	2.0 ± 1.2	1.2 ± 1.1	0.123^a^
Time since stroke (*d*)	11.2 ± 3.1	13.2 ± 2.5	0.080^a^
Study period (*d*)	23.8 ± 1.5	24.2 ± 1.8	0.539^a^

*Values are mean ± SD. MMSE, Mini-Mental State Examination; t-PA, tissue-plasminogen activator; ^*a*^Mann–Whitney *U* test; ^*b*^Fisher’s exact test.*

Patients in both the groups who did not undergo surgery during the acute phase received conservative treatment (e.g., antiplatelet therapy, brain protection therapy, antihypertensive therapy) according to individual conditions. No differences were observed between the two groups in terms of their characteristics and baseline clinical data (Table 1). The quantity of physical therapy during the intervention period, including hours spent on gait training, did not differ between the two groups (Table 2). Although the HAL group had significantly less occupational therapy and speech therapy than the CGT group (p < 0.05), it had a significantly higher walking distance (397.7 ± 178.2 vs. 187.7 ± 181.4, *p* < 0.05, Table 2). However, the actual walking time and the Modified Borg scale were not significantly different between the HAL and CGT groups at the end of the intervention.

**TABLE 2:** Various parameters related to the gait treatment period.

		**HAL group (*n* = 12)**	**Conventional group (*n* = 10)**	***P***
Waiking time (min)		14.7 ± 2.4	11.9 ± 4.4	0.159
Walking distance (m)		397.7 ± 178.2	187.4 ± 181.4	0.009
Modified Borg scale		2.6 ± 1.2	2.5 ± 1.0	0.872
Gait treatment period (d)		19.0 ± 1.3	19.0 ± 1.4	1.000
Quantity of rehabilitation	Physical therapy	15.3 ± 2.0	17.9 ± 5.0	0.346
During intervention period (h)	Occupational therapy	6.6 ± 3.7	15.4 ± 3.9	*p* < 0.001
	Speech therapy	2.7 ± 3.2*	8.5 ± 5.7	0.023
	Total amount	24.3 ± 6.8	41.9 ± 9.5	*p* < 0.001
Physical therapist’s years of experience				
		5.5 ± 4.8	2.7 ± 1.9	0.159

Values are mean ± SD. Mann–Whitney *U* test. *Ten patients in the HAL group received the speech therapy.

Significantly greater improvements in FAC scores were observed in the HAL group than in the CGT group, with differences between both the group (HAL-CON) 1.0 of median FAC score (*p* < 0.05, Table 3). However, the amount of change in the secondary outcome measures did not differ significantly between the two groups, with both groups experiencing significant improvements in each of the assessed variables (*p* < 0.05, Table 3).

**TABLE 3:** Differences within groups and between groups.

	**HAL group (*n* = 12)**			**Conventional group (*n* = 10)**			**Differences between groups HAL minus CON**	***P***
**Measures**	**Pre**	**Post**	***P***	**Pre**	**Post**	***P***		
NIHSS	3 (1.5–5.0)	2 (0–2.5)	0.003	3.5 (2.0–8.0)	2.5 (0–4)	0.016	−1	0.923
LE-FMA	18.5 (14.5–24.5)	26.5 (21.0–28.5)	0.002	24.5 (9.0–26.0)	26 (18–33)	0.005	0	0.872
FAC	2 (1–2)	4 (3–4)	0.002	2 (2–2)	3 (3–4)	0.005	1.0	0.014
6MD (m)	83.1 (29.4–154.7)	222.4 (162.3–265.5)	0.002	47.2 (34.7–63.3)	101.2 (81.1–220.4)	0.005	54.0	0.107
CWS (m/s)	0.28 (0.09–0.39)	0.50 (0.33–0.63)	0.002	0.16 (0.09–0.23)	0.30 (0.21–0.61)	0.013	0.09	0.346
Step length (m)	0.24 (0.19–0.40)	0.40 (0.35–0.46)	0.003	0.17 (0.15–0.21)	0.30 (0.21–0.43)	0.009	0	1.000
Cadence (steps/min)	53.3 (27.4–67.5)	72.4 (58.3–82.9)	0.019	57.8 (39.0–63.3)	60.4 (54.6–86.5)	0.022	3.0	0.497
BI	67.5 (55.0–75.0)	90.0 (80.0–95.0)	0.002	62.5 (55.0–70.0)	72.5 (70.0–95.0)	0.005	2.5	0.539
FIM-total	87.5 (77.0–96.0)	109.5 (102.5–116.0)	0.002	87.5 (79.0–100.0)	108.5 (99.0–121.0)	0.005	−2	0.974
FIM-motor	52.5 (43.0–64.0)	75 (67.5–82.5)	0.002	54.5 (46.0–65.0)	73.5 (64.0–86.0)	0.005	−4	0.974
FIM-cognitive	35 (34–35)	35 (35–35)	0.102	35 (29–35)	35 (35–35)	0.461	0	1.000

*Values are Median (Quartiles range). CON, conventional; NIHSS, National Institutes of Health Stroke Scale; LE-FMA, Fugl-Meyer Assessment of lower extremity; FAC, Functional Ambulation Category; 6MD, 6-minute walk distance; CWS, comfortable walking speed; BI, Barthel Index; FIM, Functional Independence Measure. Differences within groups was used Wilcoxon signed-rank test. Differences between groups was used Mann–Whitney *U* test.*

## Discussion

To the best of our knowledge, this is the first non-randomized clinical trial to examine the feasibility and efficacy of a single-leg version of HAL in patients with acute stroke. We aimed to investigate whether treatment using the motion assistance provided by a single-leg version of HAL could produce early improvements in independent walking compared to CGT in patients after acute stroke. Our preliminary data indicate that participants who received HAL treatment had a significantly greater improvement in their gait than those who received CGT based on FAC outcome measures.

Previous studies have established the safety and feasibility of HAL treatment in the early stages of stroke (Ueba et al., 2013; Nilsson et al., 2014; Ogata et al., 2015). A cohort study suggested that patients with intracerebral hemorrhage and a low Brunnstrom stage should be carefully monitored for orthostatic hypotension (Ueba et al., 2013), whereas others demonstrated that intensive gait training with HAL early after stroke is safe when delivered by experienced physical therapists as part of an inpatient rehabilitation program (Nilsson et al., 2014; Wall et al., 2015). Another retrospective study showed that there were no differences in outcomes between groups with and without HAL treatment (Ogata et al., 2015). In previous research, our group found that gait treatment with HAL improved independent walking more efficiently than CGT in patients with sub-acute stroke (Watanabe et al., 2014), and that independent walking improved significantly in the HAL group at 2 months (Watanabe et al., 2017). However, no study to date had compared the effect of HAL treatment with that of conventional treatment after acute stroke. To resolve this issue, we therefore compared gait treatment between HAL and CGT cohorts in patients after acute strokes.

A recent systematic review of the Cochrane database previously reported that patients who received electromechanical-assisted gait training and physiotherapy after a stroke had a significantly increased odds of walking independently than those who received unassisted gait training (odds ratio, 1.94; 95% confidence interval, 1.39 to 2.71; *P* < 0.001). However, there was no significant increase in the walking velocity or capacity in 6 min, with mean differences of 0.04 m/s (95% confidence interval 0.00–0.09; *P* = 0.08) and 5.84 m (95% confidence interval -16.73 to 28.40; *P* = 0.61), respectively (Mehrholz et al., 2017). We produced similar results in this study. FAC is a commonly used evaluation index in the treatment of gait acquisition after stroke. Many stroke patients experience a reduced gait capacity; therefore, this outcome measure is effective for evaluating independent walking (Holden et al., 1986; Geroin et al., 2013).

A previous study has investigated the effectiveness of robot-assisted gait training with a Lokomat vs. conventional physical therapy in patients with subacute stroke (Chang et al., 2012). This highly attractive study evaluated objective parameters such as oxygen consumption. Our research differed from this study in some major aspects. The Lokomat is a robotic-driven orthotic gait device used for posture control, body-weight support, and treadmill walking (Colombo et al., 2001). Therefore, the operating principle of this device differs from that of the HAL. In addition, the assistance provided by the Lokomat tends to become passive over time, and it is unclear how much the patient is trying to move (Hidler et al., 2009; Hornby et al., 2012; Krishnan et al., 2013). In general, repetition of motion and feedback are important factors for enhancing motor learning. Motor learning is a process of reducing differences between desired and actual behavior through performance and proactive responses to feedback (Schmidt, 1991). The lack of variability in kinematic trajectories of the lower limbs during walking in the Lokomat may limit the amount of error experienced during training, which is thought to be critical for successful motor adaptation as investigated in human studies (Emken et al., 2007; Hidler et al., 2009; Marchal-Crespo and Reinkensmeyer, 2009). In contrast, the HAL provides gait support by detecting bioelectrical signals according to the wearer’s voluntary movements (Lee and Sankai, 2005; Suzuki et al., 2007). This type of movement may enhance the recovery of the impaired neuronal network, and the interactive biofeedback approach may promote the appropriate reorganization of the neuronal network (Morishita and Inoue, 2016; Sankai and Sakurai, 2018). These benefits may facilitate the achievement of gait independence. Further study verifying the effects and mechanisms of the gait treatment with a single-leg HAL system after acute stroke is required.

A voluntary drive is essential to achieve a desired behavior in robot-assisted motor learning (Lotze, 2003). The HAL system provides motion support tailored to the wearer’s voluntary drive (Kawamoto et al., 2013), and it has been shown that the walking performance in patients wearing HAL approximates that of healthy people using HAL (Puentes et al., 2018; Tan et al., 2018). A single-leg version of HAL can give active and intensive support to the gait pattern when rehabilitating a patient after acute stroke (Nilsson et al., 2014). By assisting the knee and hip joints on the paretic side, HAL can increase the frequency or duration of gait treatment a patient can undergo in a single treatment session and over a fixed period (Sczesny-Kaiser et al., 2017). In the present study, the actual walking distance was significantly higher in the HAL group than in the CGT group, implying that more repetitions of the walking motion were possible. The HAL system allows the paralyzed side to be used without excessive fatigue and may lead to a more efficient motor learning. With these benefits, a patient could make greater gains in exercise performance over a given period compared with a patient undergoing conventional treatment. HAL also makes it possible for stroke patients with neurological disorders to repeat gait training with appropriate walking performance.

In an exploratory study, Yoshikawa et al. (2016) suggested that HAL treatment in cases of sub-acute stroke may enhance the walking speed by changing the walking pattern and asymmetry ratio in the late recovery stage. Mizukami et al. (2017) also argued that, if gait treatment with HAL can be started early, walking with an appropriate pattern can be improved and patients can avoid acquiring an incorrect or compensatory walking pattern. Early intensive HAL treatment may contribute not only to improved independent walking but also to improved walking patterns. Moreover, a recent systematic review showed that asymmetry measures provide additional information regarding the coordinative requirements for gait and can potentially be used to indicate recovery (Wonsetler and Bowden, 2017). In the future, we plan to elucidate the effects of HAL treatment on spatiotemporal parameters and asymmetry ratios.

The mechanism by which independent walking improved significantly in our HAL group is unclear. Our previous research suggests that HAL treatment may improve coordination in the lower limbs and change the pattern of muscle synergy (Puentes et al., 2018; Tan et al., 2018). Although these factors may have contributed to the outcomes observed in this pilot study, the lack of randomization will have led to the inclusion of many biases. In addition to the clinical evaluation indices, such as independent walking and walking speed, there is a need to establish new evaluation methods for motion analysis and muscle activity patterns. Improving the degree of independence of stroke patients may contribute to improvements in activities of daily living and quality of life, as well as a shortening of inpatient stays and an increase in returns to independent living. In turn, these improvements may contribute to reduced burdens on caregivers and healthcare costs, and indirectly help to solve problems with social care in Japan. Improving independent walking (i.e., reducing the amount of walking assistance) is not only important for patients, assistants, and medical staff but also for wider society (Rigby et al., 2009).

We conclude that HAL-based gait treatment may improve the independent walking ability of patients with acute stroke hemiplegia who are dependent on ambulators. However, this pilot study has several limitations that necessitate a large-scale randomized controlled trial to confirm the likely effectiveness. A major limitation was the lack of randomization, with treatment type only divided by facility. However, we did standardize both the amount of physical therapy and the breadth and type of any additional content. It was reassuring that there were no significant differences between the two groups in these factors. Another limitation was the low statistical power due to the small sample. Moreover, we could not exclude the possibility of observer bias because the same therapists implemented training and assessment without blinding to treatment allocation. In the near future, we plan to confirm our results by conducting a larger-scale randomized controlled trial with a blinded protocol and third-party evaluator.

## Data Availability Statement

The datasets for this manuscript are not publicly available because secondary use of the data is not permitted by the Clinical Research Ethics Review Board. Requests to access the datasets should be directed to AMar, aiki.marushima@md.tsukuba.ac.jp.

## Ethics Statement

The studies involving human participants were reviewed and approved by the Ethics Committee of the University of Tsukuba Hospital (H27-257). The patients/participants provided their written informed consent to participate in this study.

## Author Contributions

HW and AMar: conceptualization and writing of the original draft. MY and AMat: funding acquisition. HW, AMar, HKad, TU, YSh, SK, TH, MS, YI, MH, TT, HF, NS, and KM: methodology and project administration. AMar: review and editing. AMar, HKad, TU, YShi, SK, TH, MS, YI, MH, HT, TT, AT, HF, NS, KM, HKaw, YH, MY, YSa, EI, YM, and AMat: confirming the final manuscript.

## Conflict of Interest

HKaw is a stockholder of Cyberdyne, Inc. (Tsukuba, Japan). YSa is a stockholder and the CEO of the company, the manufacturer of the robot suit HAL. YSa’s conflict of interest is managed by board of directors of Cyberdyne and managed by the University of Tsukuba according to national university rules. The patent royalty is paid to the University of Tsukuba from Cyberdyne and a part of the royalty is paid to YSa from the University of Tsukuba according to the national university rules. This study was proposed by the authors, and Cyberdyne was not involved in the study design, in the collection, analysis, and interpretation of data, in the writing of the report, or in the decision to submit the manuscript for publication. The remaining authors declare that the research was conducted in the absence of any commercial or financial relationships that could be construed as a potential conflict of interest.
